# Single-Cell Genomic Analysis in Plants

**DOI:** 10.3390/genes9010050

**Published:** 2018-01-22

**Authors:** Yuxuan Yuan, HueyTyng Lee, Haifei Hu, Armin Scheben, David Edwards

**Affiliations:** 1 School of Biological Sciences and Institute of Agriculture, The University of Western Australia, Perth, WA 6009, Australia; yuxuan.yuan@research.uwa.edu.au (Y.Y.); jenny.lee@uwa.edu.au (H.L.); haifei.hu@research.uwa.edu.au (H.H.); armin.scheben@research.uwa.edu.au (A.S.); 2 School of Agriculture and Food Science, The University of Queensland, Brisbane, QLD 4072, Australia

**Keywords:** computational algorithms, DNA sequencing, plant, single cell analysis, technologies

## Abstract

Individual cells in an organism are variable, which strongly impacts cellular processes. Advances in sequencing technologies have enabled single-cell genomic analysis to become widespread, addressing shortcomings of analyses conducted on populations of bulk cells. While the field of single-cell plant genomics is in its infancy, there is great potential to gain insights into cell lineage and functional cell types to help understand complex cellular interactions in plants. In this review, we discuss current approaches for single-cell plant genomic analysis, with a focus on single-cell isolation, DNA amplification, next-generation sequencing, and bioinformatics analysis. We outline the technical challenges of analysing material from a single plant cell, and then examine applications of single-cell genomics and the integration of this approach with genome editing. Finally, we indicate future directions we expect in the rapidly developing field of plant single-cell genomic analysis.

## 1. Introduction

Single-cell genomic analysis is the tracking and study of single isolated cells using sequencing technologies such as whole genome sequencing (WGS) and RNA sequencing. Single-cell sequencing enables high-resolution measurements of cell-to-cell variation that is masked in conventional bulk sequencing, in which each sequencing library consists of a population of cells rather than a single cell. Single-cell analysis has been increasingly used in mammalian studies in the past decade. Single-cell DNA (scDNA) sequencing was applied for single nucleotide and copy-number variation analysis of both tumour and normal single cells [1,2,3], as well as the analysis of recombination activity in germ cells [4,5]. Single-cell RNA (scRNA) sequencing is also widely used to identify gene expression dynamics between subpopulations of cells [6].

The importance of single-cell analysis was recognised when increasing evidence showed that distinct cell types in an organism undergo specific physiological processes and contain unique mutations [7]. In humans and animals, the somatic evolution of cells [8,9] and the recombination of germlines [4] generate genomic signatures indicative of temporal developmental stages. The significance of genetic dynamics is even more emphasised in tumour cells, where genetic heterogeneity is common [10]. Similarly, plant tissues and cells are highly specialised, not only morphologically, but also biochemically and physiologically [11]. Early research has shown that the ion and metabolite distribution of individual epidermis cells in barley leaf vary depending on leaf developmental stage and light level [12]. This work highlighted the two main purposes of single-cell analysis: understanding the individuality of cell stages, and their differential response to environmental stimuli. High-resolution gene expression maps of *Arabidopsis* roots have shown that expression patterns do not always correlate with previously defined anatomical boundaries [13,14]. In shoots, isolated cell populations in the apical meristem displayed specific expression profiles, which contributed to the identification of stem cell markers [15]. Transcripts differentially expressed in cell types of the leaf epidermis were also observed in *Arabidopsis* [16], barley [17], and maize [18]. Gene expression studies have also successfully described the development and differentiation of other unique plant morphologies, such as stomatal cells [19], pollen [20,21], and female gametophytes [22]. Distinct cell-type-to-cell-type gene expression when responding to environmental stimuli suggests tight gene regulation. For example, Dinneny et al. [23] revealed that the transcriptional response of *Arabidopsis* root cells to salinity and iron deficiency are specific to the developmental stage of the cell. In a separate study, five *Arabidopsis* root cell types showed a distinct cellular response to nitrogen influx such as the cell-specific regulation of hormone signalling [24]. The assumption of the universal stress response was also rejected in other studies [25,26]. Similarly, plant defence to biotic stress is tissue-specific. For example, the transcriptional state of rice root tissues differs from leaf tissues following rice blast fungus invasion [27].

The understanding that molecular characteristics in cell types of an individual organism vary has provided new perspectives on the conclusions drawn from previous bulk sequencing studies. Single-cell genomic analysis has successfully described cancer cell states, for example, of stem cells in leukaemia patients [28] and biological developmental processes such as ageing [29]. However, technical issues, such as cell isolation difficulties [30], have delayed the use of single-cell analysis in plants. To date, two studies employed adapted protocols developed for animal systems to sequence *Arabidopsis* root cells and classify cells using clustering [31,32]. As a result, the process of root regeneration was successfully described [33]. 

Single-cell studies in plants have the potential to increase the resolution of previous studies in two major areas: (1) developmental dynamics of plant tissues to identify non-anatomical markers for important cell populations; and (2) plant stress signalling, responses, and adaptation. Here, we review the opportunities provided by plant single-cell analysis and discuss the experimental and analytical challenges that need to be addressed to maximise the scientific impact of this approach.

## 2. Challenges and Opportunities in Plant Single-Cell Analysis

**Figure 1:**
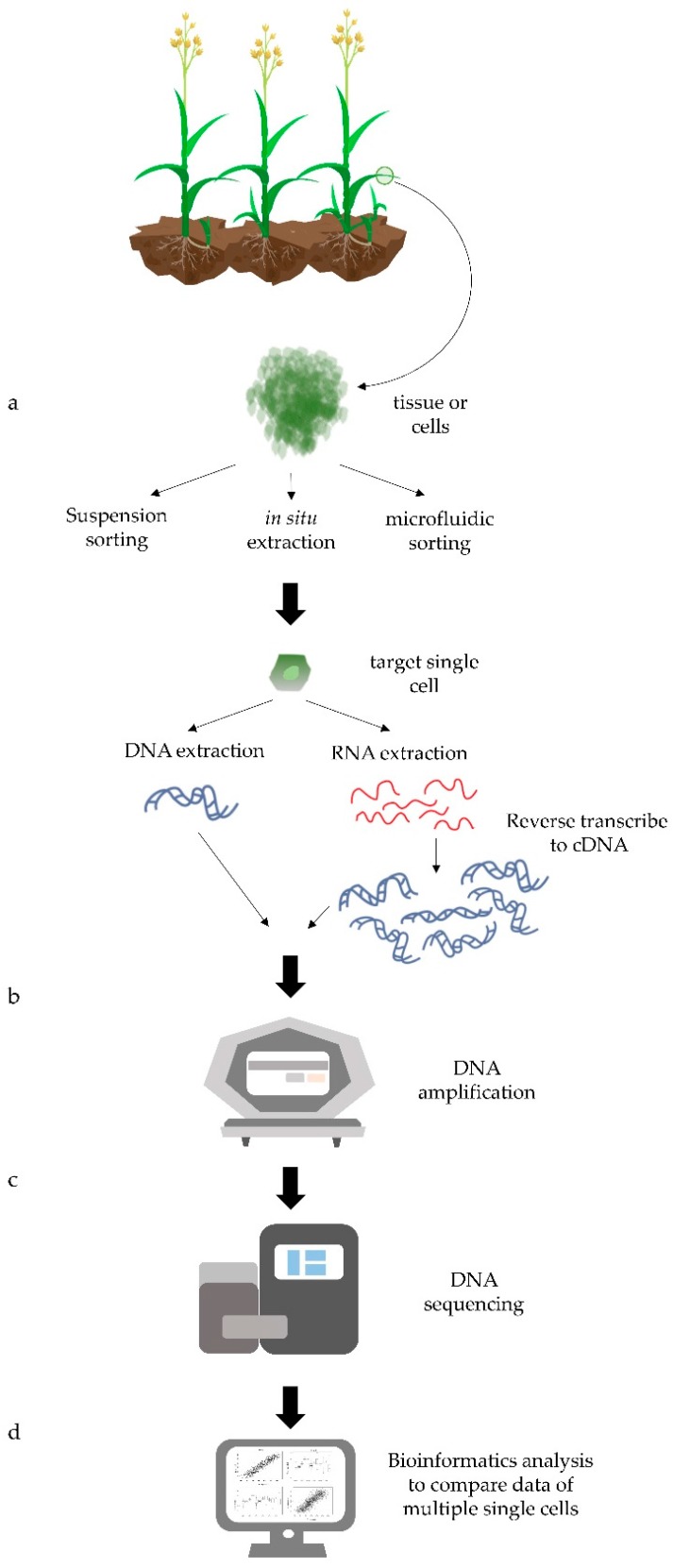
Overview of plant single-cell genomic analysis. (**a**) During single-cell preparation, target single cells are isolated in a suspension, extracted mechanically in situ, or sorted by microfluidics. After single-cell isolation, DNA or RNA is extracted. RNA is reverse transcribed to single stranded or double stranded cDNA (only double stranded cDNA shown). (**b**) To increase the amount of material for sequencing, DNA or cDNA (when studying transcripts) are amplified. (**c**) Libraries are prepared for genomic DNA or cDNA and next-generation sequencing is carried out. (**d**) Bioinformatics analysis is conducted to compare single-cell sequences and find functional variants between cells.

Single-cell genomic analysis generally comprises four steps (Figure 1): single-cell preparation, DNA amplification, next-generation sequencing, and bioinformatics analysis [34,35]. The study of single cells in plants is still in its early stages. However, recent technological advances are driving increasing interest in plant single-cell studies (Table 1 and Table 2).

### 2.1. Preparation of Single-Cell Sequencing Libraries

#### Single-Cell Isolation

To perform single-cell experiments, cells of interest first need to be isolated. However, single-cell isolation is not a trivial task, especially in complex solid tissues [35], and the development and standardisation of best practices for isolation techniques is ongoing [36]. Traditionally, the first isolation step is to macerate or remove cell walls, allowing manipulation of individual cells in a suspension [35]. Compared to animal cells, plant cells usually have rigid exteriors, which complicates isolation [30]. Macerating plant cell walls using enzymatic digestion is a feasible solution [30]. Enzymatic hydrolysis was used to isolate single cells from potato leaves [37] and apple flesh [38], indicating that pectinase is a crucial enzyme in cell isolation. However, long enzymolysis time may damage the completeness and activity of cells [38]. Later, many studies improved this method, for instance, Jia et al. [39] used cellulose digestion to obtain protoplasts from wheat leaves.

After obtaining a suspension, several approaches are used for single-cell isolation, among which are serial dilution [40], micromanipulation [7], fluorescence-activated cell sorting (FACS) [41], and optical tweezers [51]. Serial dilution is the simplest approach for isolating a single cell in a single well. During the process, cells are serially diluted to approximately one cell per microliter. However, owing to the low accuracy of serial dilution, this approach has rarely been used in recent single-cell studies. Micromanipulation is a simple and cheap method for isolating single cells such as early embryos [50]. However, micromanipulation is low-throughput, time-consuming, and has high misidentification rates [7,50]. FACS, on the other hand, is the most commonly used method to isolate individual cells based on size, granularity, and fluorescence of cells [52]. FACS has been made commercially available by companies such as BD Biosciences (San Jose, CA, USA) and Beckman Coulter (Brea, CA, USA) [53]. However, FACS requires a large number of cells in suspension (thounsands of cells), which may affect the yield of low-abundance cell subpopulations. Additionally, due to the rapid flow, cells might be damaged during FACS [7]. Optical tweezers are an alternative, using a highly focused laser beam to capture cells [7]. With the assistance of imaging-based selection, optical tweezers can isolate cells in suspension or a cell array inside a microfluidic device [6].

In addition to suspension based isolation methods, techniques such as laser microdissection (LMD), laser microdissection and pressure catapulting (LMPC), and laser capture microdissection (LCM) [42] are used to extract single cells in situ based on cellular morphology [50,54]. However, several drawbacks remain to be overcome, including low throughput, accidental slicing of cells during sectioning, UV damage to nuclei, and contamination from neighbouring cells [50,55]. Magnetic-activated cell sorting (MACS) is another commonly used single-cell isolation method. MACS is a column-based technique that isolates cells using antibodies, enzymes, or lectins to bind specific cell-surface proteins [56]. However, the high costs for the separation magnet, the columns, the antibodies, and the specific sensitivity to positively and negatively charged cell populations makes its usage far more limited than FACS [56].

More recently, microfluidic technologies have been shown to be a parallel, accurate, high-throughput, and sensitive single-cell isolation technique [43]. However, costly proprietary reagents are needed to complete the isolation when using these commercial microfluidics platforms [34]. Additionally, microfluidic platforms require uniform cell sizes [57], limiting their applicability for cell samples with varying size. Currently, microfluidics is only being used to isolate animal cells, but it is expected that it will be applied in plant cells in the near future.

### 2.2. DNA/RNA Amplification

#### 2.2.1. Whole Genome Amplification 

The process of scDNA sequencing is considerably more challenging than scRNA sequencing. The main reason for this is the error-prone nature of the DNA amplification step, which is required, as there is a limited amount of DNA that can be extracted from a single cell. For instance, a single mammalian cell generally contains less than 10 picograms (pg) of DNA [56], and plant cells may contain between <0.1 pg and >120 pg, with a low modal weight of 0.6 picograms in flowering plants [58]. As DNA sequencing generally requires over 200 nanograms of DNA, and low-input protocols still require 500 picograms to 10 nanograms of DNA (https://nanoporetech.com/products/kits; https://www.neb.com/products), scDNA sequencing requires DNA amplification. However, DNA amplification leads to nonuniform coverage, allelic dropout, and false positive mutations [57]. These technical challenges affect the results of DNA sequencing and hamper downstream analyses, complicating the discovery of real biological variation. To solve the problems related to DNA amplification, several methods have been developed. 

PCR-based methods such as linker-adapter PCR (LA-PCR) [59], interspersed repetitive sequence PCR (IRS-PCR) [60], primer extension preamplification PCR (PEP-PCR) [61], and degenerate oligonucleotide-primed PCR (DOP-PCR) [62] were initially used for scDNA amplification. However, the low genome coverage (~10%), limited production, severe amplification biases, and allelic dropout substantially limited these approaches [57]. Later, multiple displacement amplification (MDA) [44] was developed and widely used in scDNA amplification. The application of MDA is simple, generating a high genome coverage (>90%) and a low false positive rate (~10^−7^) [57]. However, nonuniform coverage and high allelic dropout rates (~31–65%) lower the sensitivity of MDA to copy number variation (CNV) [56]. To increase uniformity of coverage and decrease allelic dropout, a method called multiple annealing- and looping-based amplification cycles (MALBAC) [2] was developed. The allelic dropout rate in MALBAC is reduced to ~1%. Almost 93% of genome coverage can be amplified to 25× on average [2]. Moreover, MALBAC is particularly useful for CNV and single nucleotide variant (SNV) detection. However, the high false positive rates of MALBAC require further improvement. Another amplification method is the microwell displacement amplification system (MIDAS) [45]. MIDAS uses a massive parallel polymerase cloning method to reduce amplification bias and alleviate nonuniform coverage [63]. Compared to MDA, this method can reduce reaction volume ~1000 fold. MIDAS also reduces the template concentration required and the level of contamination [56].

#### 2.2.2. Whole Transcriptome Amplification 

Previous studies using population samples have provided insights into the distribution of gene expression levels across cells. However, the bulk cells used in RNA sequencing make it difficult to quantify gene expression in individual cells. Studies applying scRNA sequencing can shed light on variability in gene expression across cells. As the RNA material in a single cell is insufficient for scRNA sequencing, whole transcriptome amplification (WTA) is required. Compared to whole genome amplification (WGA), WTA is less challenging because the presence of multiple transcript copies reduces the dropout rate. In recent years, numerous technologies have been developed to improve WTA. Although WTA methods have improved their throughput, sensitivity, accuracy, and precision, the challenges of amplification bias and additional noise remain [64].

To characterise the transcriptome of a single cell, mRNA must be reverse-transcribed into cDNA before WTA. Prior to the use of next-generation sequencing (NGS), cDNA microarrays were applied to analyse gene expression from single cells. However, this method was less sensitive and could miss many rare but key transcripts [65]. To overcome this limitation of microarrays, in 2010, Tang et al. [66] improved the WTA method and used NGS to detect genes and splice junctions in one cell. In their method, oligo deoxythymine (dT) primers with anchor sequences were used for mRNA reverse-transcription before PCR amplification. However, this method could generate 3′-end mRNA bias mainly due to the limited length of cDNAs [67]. To alleviate this situation, a WTA method named SMART-seq [46] was developed. SMART-seq generates and amplifies full-length cDNA from single cells using Moloney murine leukaemia virus (MMLV) to perform reverse-transcription. However, the low sensitivity of SMART-seq prompted development of the improved SMART-seq2 approach [47]. SMART-seq2 enables researchers to detect gene expression differences in multiple samples, at the expense of a strong 5′-end bias.

Several in vitro transcription (IVT) methods were developed, including cell expression by linear amplification sequencing (Cel-seq) [48]. The main benefit of IVT is linear amplification, which reduces amplification bias compared to exponential amplification methods such as PCR [7]. However, the bias towards the 3′-end makes it difficult to control, which impedes the detection of the full spectrum of transcript variants [7]. To mitigate this bias, unique molecular identifiers (UMIs) are used in single-cell WTA [49]. UMIs can be implemented for quantitative scRNA sequencing with absolute molecule counts. More recently, droplet-based RNA-seq technologies have been released, including the commercial Chromium System platform (10X Genomics, Pleasanton, CA, US). Droplet-based RNA-seq technologies can differentiate the cell-of-origin of each mRNA molecule to help study single cells in complex tissues. The low level of noise generated by this approach has enabled the analysis of thousands of different cells in parallel [50].

### 2.3. Bioinformatics Analysis

Bioinformatics analysis is essential in providing biological insights and achieving the aims of single-cell experiments, such as detecting variants, quantifying gene expression, and subpopulation detection. However, conventional bioinformatics tools developed for bulk-cell genomics cannot be directly applied to single-cell sequencing data. Due to the low amount of raw genetic material, single-cell data is limited by low sequencing coverage and high amplification bias. Analytical challenges to differentiate between technical noise and true variants are further complicated by the lack of biological replicates. Furthermore, the large genome size, highly repetitive regions in plant genomes, whole genome duplications, and large amounts of gene families make bioinformatics analysis difficult [68].

To achieve a genome coverage of above 90%, 30× sequencing depth is required in single-cell sequencing, in contrast to 4× depth in bulk-cell sequencing [69]. This low coverage characteristic of single-cell sequencing data has posed difficulties in the variant calling procedure. Most bioinformatics tools employ sequence read density to call variants. Single nucleotide polymorphisms (SNPs) and small insertions/deletions with low read support are excluded in conventional bioinformatics tools. This problem is particularly evident in algorithms used to detect CNV, which strongly rely on read counts. In genome assemblies, the low coverage and heterogeneity of single-cell sequencing data also bring substantial disadvantages, leading to truncated sequences with high numbers of sequencing artefacts [35]. Recently, single-cell assemblers such as SPAdes [70] and IDBA-UD [71] have been specifically developed to overcome the challenge of amplification artefacts in single-cell sequencing and generate more precise single-cell genomic assemblies. 

In scRNA sequencing, the loss of coverage leads to low-abundance transcripts, as well as incomplete transcripts with a 3′-end bias. These transcripts affect the accurate detection of gene expression levels [72] and limit the detection of alternative splicing. For example, single blastomere cell RNA sequencing in mice produced transcripts that were approximately 3 kb shorter compared with those from conventional RNA sequencing, resulting in the loss of 36% of expressed genes [73]. Common gene expression metrics such as Fragments Per Kilobase Million/Reads Per Kilobase Million (FPKM/RPKM) do not address these 3′-end biases [69] and thus have a limited application for scRNA sequencing. To overcome the biased quantification of gene expression resulting from incomplete transcript amplification, an unbiased metric for gene expression is required. For instance, a novel synthetic statistical approach provided by Korthauer et al. [74] allows an unbiased characterisation of differences in transcript expression distribution. By utilising a Bayesian modelling framework, this novel approach can characterise differences of expression in scRNA sequencing experiments and identify biological heterogeneity with multi-modal expression with differential distributions. A second strategy is to normalise the differences in the single-cell transcripts. In this case, gene expression levels are quantified based on the normalised RNA sequencing data instead of the full-length RNA transcripts [72]. Finally, a third method for characterising gene expression is to apply unbiased clustering methods such as principal component analysis (PCA). PCA is a nonlinear dimensionality-reduction method that effectively clusters similar cells in two or three dimensions [75]. In addition, machine learning approaches have become an effective tool to addresses low sequencing coverage and amplified artefacts in scRNA sequencing. For example, Wang et al. [76] developed a machine learning algorithm called single-cell interpretation via multiple-kernel learning (SIMLR). The authors reanalysed seven representative scRNA sequencing datasets with random amplification biases, obtaining a higher clustering sensitivity and accuracy. Lin et al. [77] introduced a neural network approach to analyse scRNA sequencing data. The neural network enables simplification of scRNA sequencing data by reducing data dimension representation and accurate prediction of cell type or state through querying a database with thousands of single-cell transcriptome profiles.

The amplification bias in single-cell sequencing is another challenge for bioinformatics tools tailored for bulk-cell sequencing. In CNV detection, the amplification bias in scDNA sequencing can lead to the generation of multiple reads that obscure the correct prediction of CNVs. It is therefore necessary to examine the amplification bias to identify the associated pattern of candidate CNVs, including GC content, variant position, and repeat sequences [69]. This additional information can be incorporated into novel CNV identification algorithms, which combine a synthetic-normal-based DNA sequencing tool (SynthEx) with allele-specific copy number analysis (ASCN) [78], to address amplification bias and reduce unexpected variations in single-cell sequencing data [79]. Other newly developed single-cell CNV detection algorithms, which include GC correction [80], binary segmentation [81] and rank segmentation [82], have also enabled high detection accuracy at the base-pair level. 

Amplification bias also leads to a large proportion of false-positive SNP calling [35]. Zong et al. [2] indicated that errors in amplification during single-cell amplification could cause around one in 20 false-positive SNPs using the Genome Analysis Toolkit (GATK) [83]. Additionally, amplification failure of one or both alleles result in a high rate of allelic dropout [84], which contributes to the phenomenon of missing heterozygosity. Zong et al. [2] estimated that the value of allelic dropout could reach up to 60% for scDNA sequencing, which leads to inaccurate SNP calling. To reduce the false-positive SNPs produced by the amplification bias, two common strategies are employed. Firstly, SNPs from bulk DNA samples can be used as a reference to filter out the false-positive results [35]. Secondly, SNPs can be verified within two to three different single-cell samples, which can effectively reduce the false-positive variants introduced by amplification errors [69]. Nevertheless, no specific research has been carried out to investigate the actual number of single-cell samples that are required to validate SNPs in interrogated genomic regions.To avoid allelic dropout, one possible strategy is to apply a further filtering algorithm that identifies and removes noisy SNPs based on control groups [85].

## 3. Future Directions of Single-Cell Analysis in Plants

### 3.1. Applications of Single-Cell Analysis in Plants

Prior to the developments of modern single-cell technology, specific cell types such as root hairs [86,87,88], cotton fibres [89], and trichomes [90] served as early single-cell-type models due to their easy isolation. When compared to bulk-cells studies, these single-cell-type models increased the resolution of our understanding in cellular processes and differentiation of plant roots, cell walls, and shoot epidermal hair. For example, despite being morphologically recognised as leaf trichomes, gene expression profiles during secondary wall cellulose synthesis in cotton fibres resembled sclerenchyma cells [89,91]. In another example, transcriptomes of root hair single cells isolated from soybean only contain 25% of the transcription factors found in whole root transcriptome studies [88].

Plant cells show high developmental plasticity, and differentiated somatic plant cells can be stimulated to form embryos in culture [92]. However, it remains unclear whether plant cell-fate regulation is a lineage-dependent mechanism, as in animals [93], or based on cell relative position [94], or a mix of both [95]. Single-cell analysis can be used to map individual cell stage from initial to differentiated, therefore shedding light on regeneration mechanisms, cell-fate regulation, and totipotency in general. Protocols for single-cell lineage tracing were established in animal and human studies [96], and could be adapted for use in plant analysis. Recent single-cell analysis of *Arabidopsis* roots showed that multiple cell types could rapidly reconstitute stem cells by replaying the patterns of embryogenesis [33], therefore supporting the notion of a decentralised stem cell control system [97]. Single-cell transcriptomics can further contribute to the identification of critical genes in regeneration, which can be tracked and used as markers for developmental studies. 

Due to environmental variation, stress tolerance of plants has always been of great interest in both disease resistance as well as trait improvement for crop breeding. Whole tissue bulk material is widely used to understand stress signalling in plants (examples in *Arabidopsis* [98,99,100]) and to detect markers such as nucleotide polymorphisms (e.g., in soybean flowering [101]) and CNVs (e.g., in rice grain size [102]) as the basis of crop breeding programs. However, as stress regulation is cell type-specific [103], bulk tissue analysis diluted plant response signals and overlooked cell-type-specific structural variation. Advances in single-cell sequencing can thus offer novel insights into stress adaptation in plants, particularly for modelling gene regulatory networks. For example, plant hormones are the key mediators of stress response [104], yet the interactions between hormone signalling pathways are poorly understood [105]. A recent analysis showed that interactions between hormones directly manipulate tissue formation and patterning using single-cell information [33]. This work could be applied to model hormone signalling networks in stress responses, such as dissecting the conflicting evidence of ethylene as a positive or negative regulator during high salinity stress in different species at different developmental stages [106], as well as the ethylene-jasmonate-abscisic acid crosstalks [107,108,109]. Single-cell analysis can also detect novel regulatory processes. One example is the identification of new rhizobial infection-related genes and novel processes in *Medicago* root hair that were previously undetected in bulk-cell whole-root studies [110]. There is also increasing evidence of the regulation of stress response by alternative splicing [111], for example alternative isoforms of resistance genes regulate defence against tobacco mosaic virus [112]; alternative splicing occurs as a result of temperature-induced stress in *Arabidopsis* [113]. As gene isoforms were also shown to be allocated to different cell types [114], single-cell analysis has the potential to mark and track alternative transcripts following developmental stages and stimuli.

Applying single-cell analysis in plants can discover unknown cell types through deconvoluting heterogeneous cell populations by unbiased identification of biological variation between adjacent cell states. The current description of plant cell states is still widely based on morphology and known markers [30]. Signatures of rare subpopulations can be detected through single-cell technology, as demonstrated in human T cells [75]. In addition, the development of single-cell analysis in plants will contribute to the collection of physiologically-based markers and serve as a foundation for cell type marking in future work.

### 3.2. Integration with Genome Editing

The genome editing technology clustered regularly interspaced short palindromic repeat (CRISPR)/CRISPR-associated protein (Cas) enables the prediction of gene function using highly parallel pooled mutation screens [115,116]. Current CRISPR/Cas-pooled screens suffer from limited resolution to study individual mutant genotypes and associated transcriptomes, leading to high false-positive and false-negative results [72,117]. Recently, this limitation of CRISPR/Cas screens has been overcome by combining them with scRNA sequencing in approaches referred to as CRISPR-seq [118], CROP-seq [119], and Perturb-seq [120]. These approaches allow detection of the transcriptional effects of multiple gene disruption events in hundreds of thousands of single cells. Although these techniques have only been applied in mammalian cells, they have potential to shed light on gene functions and regulatory pathways in plants. In particular, model and crop plants are suitable for these genetic screens, as high-quality genome assemblies and knowledge of target genes is an important prerequisite. 

CRISPR-seq and related techniques rely on a guide RNA (gRNA) vector with a unique barcode that can be detected in scRNA sequencing, a massively parallel scRNA sequencing assay, and a bioinformatics pipeline for obtaining gRNAs from single-cell transcriptomes and analysing the generated transcriptional profiles. CRISPR-seq requires compartmentalisation of each guide RNA (gRNA) and its biological signal in a single cell [119]. A gRNA is transferred into one single cell in the pool, inducing a specific knockout in a targeted gene. By measuring gRNA in each cell and its corresponding transcriptome, scRNA sequencing can directly detect precise gene expression levels of each targeted gene knockout on a large scale of cells. For example, Jaitin et al. [118] were able measure the expression of single gRNAs at 50,000 cells per well in a 500 μL culture solution. Compared with the classical pooled screening method (gene knockout followed by transcriptome analysis), CRISPR-seq combines gene knockout and expression analysis in one step to provide a simpler, cheaper, more flexible, and more efficient method to study biological mechanisms in various cell states or cell types [118]. Diego et al. [118] utilised CRISPR-seq to investigate the regulatory mechanism of myeloid cells during cell differentiation and the expression level of significant developmental and immune-related regulators. They indicated that the transcription factors CEBPB and IRF8 play opposing roles in regulating development of monocyte/macrophage versus dendritic cell lineages. Wang et al. [121] also applied CRISPR-seq to identify significant genes required for mammalian cell proliferation and formation of cancer cells. 

When the difficulties of isolating single plant cells are overcome, CRISPR-seq will become a new generation genome editing tool improving knowledge on plant genetics, with a potentially substantial impact on plant breeding. CRISPR-seq enables sequential knockout of target genes in crop cells, allowing large-scale gene function analysis across cell lineages. For instance, CRISPR-seq might be applied for studying the expression level of regulatory factors such as LEC1, WUS, and ODP2 during cell proliferation and differentiation [122]. A better undererstanding of these regulatory factors could help induce the formation of somatic embryos from plant tissue cultures to accelerate the breeding cycle [122]. With genome editing now allowing precise modification of DNA and RNA [123], tools to assay plant cells for suitable functional editing targets will become increasingly important.

### 3.3. Data Repository for Plant Cells

As the study of single cells is still evolving, protocols used in WGA and WTA at the moment are diverse and difficult to standardise [34]. Algorithms developed for data extraction and compilation are different. In single-cell studies, the amount of genome and transcriptome data generated poses a potential challenge for data storage and sharing. To efficiently document each single-cell experiment, data repositories are required and should be able to categorise each data format and make data reusable, shareable, and comparable. To achieve this, proper data management and novel algorithms are needed to ensure users track experimental parameters and allow upload and download of plant single-cell data. 

In the study of bulk cells or tissues, data repositories, such as the National Center for Biotechnology Information (NCBI), provide a good example for data storage and management. However, for single-cell sequencing data, although NCBI has already provided a similar service, it has missed the importance and demand for experimental metadata such as molecular information. In the near future, comprehensive data repositories for single cells are expected. Some standardised experimental data formats similar to the established sequence format FASTQ or the alignment map format BAM are also needed to make the study of single cells more robust. 

## 4. Conclusions

Single-cell genomic analysis provides novel solutions for studying cells that play important roles in system behaviour, tissue development, regeneration, and repair. By studying biological diversity in plant cells or tissues, the development of plant organs and the response of plants to environmental stress will be better understood. Combined with gene editing technologies and modelling of regulatory networks for target discovery, single-cell sequencing will boost crop improvement. Although challenges remain in single-cell preparation, DNA/RNA amplification, DNA sequencing, and bioinformatics analysis, the rapid evolution of single-cell technologies is expected to play an important role in feeding the world by helping to breed high-yielding and stress-tolerant elite cultivars.

## Figures and Tables

**Table 1:** Comparison of selected single-cell isolation approaches.

Isolation Approach		Accuracy	Cell Material Required	Throughput	Challenges
suspension	Serial dilution [40]	low	high	low	low accuracy
	micromanipulation [7]	moderate	low	low	low-throughtput; time-consuming; high misidentification rates
	fluorescence-activated cell sorting (FACS) [41]	high	high	high	requires a large number of cells; may affect the yield of low-abundance cell subpopulations; may damage cells
in situ	laser microdissection (LMD)	moderate	high	Low	low throughput; accidental slicing of cells; UV damage to nuclei and contamination from neighbouring cells
	laser microdissection and pressure catapulting (LMPC)	moderate	high	Low	
	laser capture microdissection (LCM) [42]	moderate	high	Low	
microfluidics	microfluidics [43]	high	moderate to high	high	high cost; needs uniform cell sizes

**Table 2:** Comparison of selected nucleic acid amplification approaches for single-cell sequencing.

Nucleic Acid	Amplification Approach	Amount of Nucleic Acid Input	Genomic Coverage	Uniformity of Coverage	Dropout Rate	Challenges
DNA	PCR	moderate	low	low	high	low genome coverage; limited yield; severe amplification biases and allelic dropout
	multiple displacement amplification (MDA) [44]	moderate	high	low	high	Nonuniform coverage; high allelic dropout rates
	microwell displacement amplification system (MIDAS) [45]	low	high	high	low	relatively low efficiency of amplification; amplicon extraction is performed manually; cross contamination between wells
RNA	SMART-seq [46] and SMART-seq2 [47]	moderate	high	moderate	low	low sensitivity; high 5′-end bias
	in vitro transcription using cell expression by linear amplification sequencing (Cel-seq) [48]	moderate	high	moderate	low	high 3′-end bias
	unique molecular identifiers (UMIs) [49]	moderate	high	high	low	number of UMIs can be overestimated; cell doublets
	droplet-based Chromium System platform [50]	high	high	high	low	commerical libraries are needed

## References

[1] Baslan T., Kendall J., Rodgers L., Cox H., Riggs M., Stepansky A., Troge J., Ravi K., Esposito D., Lakshmi B. (2012). Genome-wide copy number analysis of single cells. Nat. Protoc..

[2] Zong C., Lu S., Chapman A.R., Xie X.S. (2012). Genome-wide detection of single-nucleotide and copy-number variations of a single human cell. Science.

[3] Navin N., Kendall J., Troge J., Andrews P., Rodgers L., McIndoo J., Cook K., Stepansky A., Levy D., Esposito D. (2011). Tumour evolution inferred by single-cell sequencing. Nature.

[4] Wang J., Fan H.C., Behr B., Quake S.R. (2012). Genome-wide single-cell analysis of recombination activity and de novo mutation rates in human sperm. Cell.

[5] Lu S., Zong C., Fan W., Yang M., Li J., Chapman A.R., Zhu P., Hu X., Xu L., Yan L. (2012). Probing meiotic recombination and aneuploidy of single sperm cells by whole-genome sequencing. Science.

[6] Saliba A.E., Westermann A.J., Gorski S.A., Vogel J. (2014). Single-cell RNA-Seq: Advances and future challenges. Nucleic Acids Res..

[7] Shapiro E., Biezuner T., Linnarsson S. (2013). Single-cell sequencing-based technologies will revolutionize whole-organism science. Nat. Rev. Genet..

[8] Frumkin D., Wasserstrom A., Kaplan S., Feige U., Shapiro E. (2005). Genomic variability within an organism exposes its cell lineage tree. PLoS Comput. Biol..

[9] Schatz D.G., Swanson P.C. (2011). V(d)j recombination: Mechanisms of initiation. Annu. Rev. Genet..

[10] Yates L.R., Campbell P.J. (2012). Evolution of the cancer genome. Nat. Rev. Genet..

[11] Nelson T., Gandotra N., Tausta S.L. (2008). Plant cell types: Reporting and sampling with new technologies. Curr. Opin. Plant Biol..

[12] Fricke W., Pritchard J., Leigh R.A., Tomos A.D. (1994). Cells of the upper and lower epidermis of barley (*Hordeum vulgare* L.) leaves exhibit distinct patterns of vacuolar solutes. Plant Physiol..

[13] Birnbaum K., Shasha D.E., Wang J.Y., Jung J.W., Lambert G.M., Galbraith D.W., Benfey P.N. (2003). A gene expression map of the *Arabidopsis* root. Science.

[14] Brady S.M., Orlando D.A., Lee J.Y., Wang J.Y., Koch J., Dinneny J.R., Mace D., Ohler U., Benfey P.N. (2007). A high-resolution root spatiotemporal map reveals dominant expression patterns. Science.

[15] Yadav R.K., Girke T., Pasala S., Xie M., Reddy G.V. (2009). Gene expression map of the *Arabidopsis* shoot apical meristem stem cell niche. Proc. Natl. Acad. Sci. USA.

[16] Lieckfeldt E., Simon-Rosin U., Kose F., Zoeller D., Schliep M., Fisahn J. (2008). Gene expression profiling of single epidermal, basal and trichome cells of *Arabidopsis thaliana*. J. Plant Physiol..

[17] Lu C., Koroleva O.A., Farrar J.F., Gallagher J., Pollock C.J., Tomos A.D. (2002). Rubisco small subunit, chlorophyll a/b-binding protein and sucrose:fructan-6-fructosyl transferase gene expression and sugar status in single barley leaf cells in situ. Cell type specificity and induction by light. Plant Physiol..

[18] Nakazono M., Qiu F., Borsuk L.A., Schnable P.S. (2003). Laser-capture microdissection, a tool for the global analysis of gene expression in specific plant cell types: Identification of genes expressed differentially in epidermal cells or vascular tissues of maize. Plant Cell.

[19] Adrian J., Chang J., Ballenger C.E., Bargmann B.O., Alassimone J., Davies K.A., Lau O.S., Matos J.L., Hachez C., Lanctot A. (2015). Transcriptome dynamics of the stomatal lineage: Birth, amplification, and termination of a self-renewing population. Dev. Cell.

[20] Becker J.D., Boavida L.C., Carneiro J., Haury M., Feijo J.A. (2003). Transcriptional profiling of *Arabidopsis* tissues reveals the unique characteristics of the pollen transcriptome. Plant Physiol..

[21] Honys D., Twell D. (2003). Comparative analysis of the *Arabidopsis* pollen transcriptome. Plant Physiol..

[22] Schmid M.W., Schmidt A., Grossniklaus U. (2015). The female gametophyte: An emerging model for cell type-specific systems biology in plant development. Front. Plant Sci..

[23] Dinneny J.R., Long T.A., Wang J.Y., Jung J.W., Mace D., Pointer S., Barron C., Brady S.M., Schiefelbein J., Benfey P.N. (2008). Cell identity mediates the response of *Arabidopsis* roots to abiotic stress. Science.

[24] Gifford M.L., Dean A., Gutierrez R.A., Coruzzi G.M., Birnbaum K.D. (2008). Cell-specific nitrogen responses mediate developmental plasticity. Proc. Natl. Acad. Sci. USA.

[25] Iyer-Pascuzzi A.S., Jackson T., Cui H., Petricka J.J., Busch W., Tsukagoshi H., Benfey P.N. (2011). Cell identity regulators link development and stress responses in the *Arabidopsis* root. Dev. Cell.

[26] Kiegle E., Moore C.A., Haseloff J., Tester M.A., Knight M.R. (2000). Cell-type-specific calcium responses to drought, salt and cold in the *Arabidopsis* root. Plant J..

[27] Marcel S., Sawers R., Oakeley E., Angliker H., Paszkowski U. (2010). Tissue-adapted invasion strategies of the rice blast fungus *Magnaporthe oryzae*. Plant Cell.

[28] Giustacchini A., Thongjuea S., Barkas N., Woll P.S., Povinelli B.J., Booth C.A.G., Sopp P., Norfo R., Rodriguez-Meira A., Ashley N. (2017). Single-cell transcriptomics uncovers distinct molecular signatures of stem cells in chronic myeloid leukemia. Nat. Med..

[29] Kowalczyk M.S., Tirosh I., Heckl D., Rao T.N., Dixit A., Haas B.J., Schneider R.K., Wagers A.J., Ebert B.L., Regev A. (2015). Single-cell RNA-seq reveals changes in cell cycle and differentiation programs upon aging of hematopoietic stem cells. Genome Res..

[30] Efroni I., Birnbaum K.D. (2016). The potential of single-cell profiling in plants. Genome Biol..

[31] Brennecke P., Anders S., Kim J.K., Kolodziejczyk A.A., Zhang X., Proserpio V., Baying B., Benes V., Teichmann S.A., Marioni J.C. (2013). Accounting for technical noise in single-cell RNA-seq experiments. Nat. Methods.

[32] Efroni I., Ip P.L., Nawy T., Mello A., Birnbaum K.D. (2015). Quantification of cell identity from single-cell gene expression profiles. Genome Biol..

[33] Efroni I., Mello A., Nawy T., Ip P.L., Rahni R., DelRose N., Powers A., Satija R., Birnbaum K.D. (2016). Root regeneration triggers an embryo-like sequence guided by hormonal interactions. Cell.

[34] Chen X., Love J.C., Navin N.E., Pachter L., Stubbington M.J., Svensson V., Sweedler J.V., Teichmann S.A. (2016). Single-cell analysis at the threshold. Nat. Biotechnol..

[35] Gawad C., Koh W., Quake S.R. (2016). Single-cell genome sequencing: Current state of the science. Nat. Rev. Genet..

[36] Eberwine J., Sul J.Y., Bartfai T., Kim J. (2014). The promise of single-cell sequencing. Nat. Methods.

[37] Zhang N., Huai-Jun S.I., Wang D. (2004). Techniques on isolation of single cells in potato. Chin. Potato.

[38] Guan Y., Qu H. (2017). A rapid method for isolating single cells from apple flesh. Hortic. Plant J..

[39] Jia X., Zhang X., Qu J., Han R. (2016). Optimization conditions of wheat mesophyll protoplast isolation. Agric. Sci..

[40] Ham R.G. (1965). Clonal growth of mammalian cells in a chemically defined, synthetic medium. Proc. Natl. Acad. Sci. USA.

[41] Spangrude G.J., Heimfeld S., Weissman I.L. (1988). Purification and characterization of mouse hematopoietic stem cells. Science.

[42] Emmert-Buck M.R., Bonner R.F., Smith P.D., Chuaqui R.F., Zhuang Z., Goldstein S.R., Weiss R.A., Liotta L.A. (1996). Laser capture microdissection. Science.

[43] Whitesides G.M. (2006). The origins and the future of microfluidics. Nature.

[44] Dean F.B., Hosono S., Fang L., Wu X., Faruqi A.F., Bray-Ward P., Sun Z., Zong Q., Du Y., Du J. (2002). Comprehensive human genome amplification using multiple displacement amplification. Proc. Natl. Acad. Sci. USA.

[45] Gole J., Gore A., Richards A., Chiu Y.J., Fung H.L., Bushman D., Chiang H.I., Chun J., Lo Y.H., Zhang K. (2013). Massively parallel polymerase cloning and genome sequencing of single cells using nanoliter microwells. Nat. Biotechnol..

[46] Zhu Y.Y., Machleder E.M., Chenchik A., Li R., Siebert P.D. (2001). Reverse transcriptase template switching: A smart approach for full-length cDNA library construction. BioTechniques.

[47] Picelli S., Bjorklund A.K., Faridani O.R., Sagasser S., Winberg G., Sandberg R. (2013). SMART-Seq2 for sensitive full-length transcriptome profiling in single cells. Nat. Methods.

[48] Hashimshony T., Wagner F., Sher N., Yanai I. (2012). Cel-seq: Single-cell RNA-Seq by multiplexed linear amplification. Cell Rep..

[49] Islam S., Zeisel A., Joost S., La Manno G., Zajac P., Kasper M., Lonnerberg P., Linnarsson S. (2014). Quantitative single-cell RNA-Seq with unique molecular identifiers. Nat. Methods.

[50] Wang J., Song Y. (2017). Single cell sequencing: A distinct new field. Clin. Transl. Med..

[51] Landry Z.C., Giovanonni S.J., Quake S.R., Blainey P.C. (2013). Optofluidic cell selection from complex microbial communities for single-genome analysis. Methods Enzymol..

[52] Lindstrom S., Andersson-Svahn H. (2010). Overview of single-cell analyses: Microdevices and applications. Lab. Chip..

[53] Navin N., Hicks J. (2011). Future medical applications of single-cell sequencing in cancer. Genome Med..

[54] Misra B.B., Assmann S.M., Chen S. (2014). Plant single-cell and single-cell-type metabolomics. Trends Plant Sci..

[55] Navin N.E. (2014). Cancer genomics: One cell at a time. Genome Biol..

[56] Hu P., Zhang W., Xin H., Deng G. (2016). Single cell isolation and analysis. Front. Cell Dev. Biol..

[57] Wang Y., Navin N.E. (2015). Advances and applications of single-cell sequencing technologies. Mol. Cell.

[58] Gregory T.R. (2005). The *C*-value enigma in plants and animals: A review of parallels and an appeal for partnership. Ann. Bot..

[59] Sugimoto K., Gordon S.P., Meyerowitz E.M. (2011). Regeneration in plants and animals: Dedifferentiation, transdifferentiation, or just differentiation?. Trends Cell Biol..

[60] McCarthy L., Hunter K., Schalkwyk L., Riba L., Anson S., Mott R., Newell W., Bruley C., Bar I., Ramu E. (1995). Efficient high-resolution genetic mapping of mouse interspersed repetitive sequence PCR products, toward integrated genetic and physical mapping of the mouse genome. Proc. Natl. Acad. Sci. USA.

[61] Birnbaum K.D. (2016). How many ways are there to make a root?. Curr. Opin. Plant Biol..

[62] Hossain M.S., Joshi T., Stacey G. (2015). System approaches to study root hairs as a single cell plant model: current status and future perspectives. Front. Plant Sci..

[63] Yalcin D., Hakguder Z.M., Otu H.H. (2016). Bioinformatics approaches to single-cell analysis in developmental biology. Mol. Hum. Reprod..

[64] Kalisky T., Oriel S., Bar-Lev T.H., Ben-Haim N., Trink A., Wineberg Y., Kanter I., Gilad S., Pyne S. (2017). A brief review of single-cell transcriptomic technologies. Brief. Funct. Genom..

[65] Brady G., Iscove N.N. (1993). Construction of cDNA libraries from single cells. Methods Enzymol..

[66] Tang F., Barbacioru C., Nordman E., Li B., Xu N., Bashkirov V.I., Lao K., Surani M.A. (2010). RNA-Seq analysis to capture the transcriptome landscape of a single cell. Nat. Protoc..

[67] Liang J., Cai W., Sun Z. (2014). Single-cell sequencing technologies: current and future. J. Genet. Genom..

[68] Yuan Y., Bayer P.E., Batley J., Edwards D. (2017). Improvements in genomic technologies: Application to crop genomics. Trends Biotechnol..

[69] Ning L., Liu G., Li G., Hou Y., Tong Y., He J. (2014). Current challenges in the bioinformatics of single cell genomics. Front. Oncol..

[70] Bankevich A., Nurk S., Antipov D., Gurevich A.A., Dvorkin M., Kulikov A.S., Lesin V.M., Nikolenko S.I., Pham S., Prjibelski A.D. (2012). Spades: A new genome assembly algorithm and its applications to single-cell sequencing. J. Comput. Biol..

[71] Peng Y., Leung H.C., Yiu S.M., Chin F.Y. (2012). Idba-ud: A de novo assembler for single-cell and metagenomic sequencing data with highly uneven depth. Bioinformatics.

[72] Stegle O., Teichmann S.A., Marioni J.C. (2015). Computational and analytical challenges in single-cell transcriptomics. Nat. Rev. Genet..

[73] Tang F., Barbacioru C., Wang Y., Nordman E., Lee C., Xu N., Wang X., Bodeau J., Tuch B.B., Siddiqui A. (2009). mRNA-Seq whole-transcriptome analysis of a single cell. Nat. Methods.

[74] Korthauer K.D., Chu L.F., Newton M.A., Li Y., Thomson J., Stewart R., Kendziorski C. (2016). A statistical approach for identifying differential distributions in single-cell RNA-Seq experiments. Genome Biol..

[75] Buettner F., Natarajan K.N., Casale F.P., Proserpio V., Scialdone A., Theis F.J., Teichmann S.A., Marioni J.C., Stegle O. (2015). Computational analysis of cell-to-cell heterogeneity in single-cell RNA-sequencing data reveals hidden subpopulations of cells. Nat. Biotechnol..

[76] Wang B., Zhu J., Pierson E., Ramazzotti D., Batzoglou S. (2017). Visualization and analysis of single-cell RNA-Seq data by kernel-based similarity learning. Nat. Methods.

[77] Lin C., Jain S., Kim H., Bar-Joseph Z. (2017). Using neural networks for reducing the dimensions of single-cell RNA-Seq data. Nucleic Acids Res..

[78] Silva G.O., Siegel M.B., Mose L.E., Parker J.S., Sun W., Perou C.M., Chen M. (2017). Synthex: A synthetic-normal-based DNA sequencing tool for copy number alteration detection and tumor heterogeneity profiling. Genome Biol..

[79] Sasagawa Y., Nikaido I., Hayashi T., Danno H., Uno K.D., Imai T., Ueda H.R. (2013). Quartz-seq: A highly reproducible and sensitive single-cell RNA sequencing method, reveals non-genetic gene-expression heterogeneity. Genome Biol..

[80] Zhao M., Wang Q., Wang Q., Jia P., Zhao Z. (2013). Computational tools for copy number variation (CNV) detection using next-generation sequencing data: features and perspectives. BMC Bioinform..

[81] Shi Y., Majewski J. (2013). Fishingcnv: A graphical software package for detecting rare copy number variations in exome-sequencing data. Bioinformatics.

[82] Mayrhofer M., Viklund B., Isaksson A. (2016). Rawcopy: Improved copy number analysis with Affymetrix arrays. Sci. Rep..

[83] McKenna A., Hanna M., Banks E., Sivachenko A., Cibulskis K., Kernytsky A., Garimella K., Altshuler D., Gabriel S., Daly M. (2010). The Genome Analysis Toolkit: A map reduce framework for analyzing next-generation DNA sequencing data. Genome Res..

[84] Piyamongkol W., Bermudez M.G., Harper J.C., Wells D. (2003). Detailed investigation of factors influencing amplification efficiency and allele drop-out in single cell PCR: implications for preimplantation genetic diagnosis. Mol. Hum. Reprod..

[85] Wills Q.F., Mead A.J. (2015). Application of single-cell genomics in cancer: promise and challenges. Hum. Mol. Genet..

[86] Kwasniewski M., Janiak A., Mueller-Roeber B., Szarejko I. (2010). Global analysis of the root hair morphogenesis transcriptome reveals new candidate genes involved in root hair formation in barley. J. Plant Physiol..

[87] Lan P., Li W., Lin W.D., Santi S., Schmidt W. (2013). Mapping gene activity of *Arabidopsis* root hairs. Genome Biol..

[88] Libault M., Farmer A., Brechenmacher L., Drnevich J., Langley R.J., Bilgin D.D., Radwan O., Neece D.J., Clough S.J., May G.D. (2010). Complete transcriptome of the soybean root hair cell, a single-cell model, and its alteration in response to *Bradyrhizobium japonicum* infection. Plant Physiol..

[89] Haigler C.H., Singh B., Wang G., Zhang D., Paterson A.H. (2009). Genomics of cotton fiber secondary wall deposition and cellulose biogenesis. Genetics and Genomics of Cotton.

[90] Hulskamp M. (2004). Plant trichomes: A model for cell differentiation. Nat. Rev. Mol. Cell Biol..

[91] Betancur L., Singh B., Rapp R.A., Wendel J.F., Marks M.D., Roberts A.W., Haigler C.H. (2010). Phylogenetically distinct cellulose synthase genes support secondary wall thickening in *Arabidopsis* shoot trichomes and cotton fiber. J. Integr. Plant Biol..

[92] Nabors M.W. (2004). Introduction to Botany.

[93] Kragl M., Knapp D., Nacu E., Khattak S., Maden M., Epperlein H.H., Tanaka E.M. (2009). Cells keep a memory of their tissue origin during axolotl limb regeneration. Nature.

[94] Kidner C., Sundaresan V., Roberts K., Dolan L. (2000). Clonal analysis of the *Arabidopsis* root confirms that position, not lineage, determines cell fate. Planta.

[95] Yu Q., Li P., Liang N., Wang H., Xu M., Wu S. (2017). Cell-fate specification in *Arabidopsis* roots requires coordinative action of lineage instruction and positional reprogramming. Plant Physiol..

[96] Woodworth M.B., Girskis K.M., Walsh C.A. (2017). Building a lineage from single cells: Genetic techniques for cell lineage tracking. Nat. Rev. Genet..

[97] Rahni R., Efroni I., Birnbaum K.D. (2016). A case for distributed control of local stem cell behavior in plants. Dev. Cell.

[98] Coolen S., Proietti S., Hickman R., Davila Olivas N.H., Huang P.P., Van Verk M.C., Van Pelt J.A., Wittenberg A.H., De Vos M., Prins M. (2016). Transcriptome dynamics of *Arabidopsis* during sequential biotic and abiotic stresses. Plant J..

[99] Rasmussen S., Barah P., Suarez-Rodriguez M.C., Bressendorff S., Friis P., Costantino P., Bones A.M., Nielsen H.B., Mundy J. (2013). Transcriptome responses to combinations of stresses in *Arabidopsis*. Plant Physiol..

[100] Zeller G., Henz S.R., Widmer C.K., Sachsenberg T., Ratsch G., Weigel D., Laubinger S. (2009). Stress-induced changes in the *Arabidopsis thaliana* transcriptome analyzed using whole-genome tiling arrays. Plant J..

[101] Xia Z., Watanabe S., Yamada T., Tsubokura Y., Nakashima H., Zhai H., Anai T., Sato S., Yamazaki T., Lu S. (2012). Positional cloning and characterization reveal the molecular basis for soybean maturity locus e1 that regulates photoperiodic flowering. Proc. Natl. Acad. Sci. USA.

[102] Wang Y., Xiong G., Hu J., Jiang L., Yu H., Xu J., Fang Y., Zeng L., Xu E., Xu J. (2015). Copy number variation at the gl7 locus contributes to grain size diversity in rice. Nat. Genet..

[103] Nouri M.Z., Moumeni A., Komatsu S. (2015). Abiotic stresses: Insight into gene regulation and protein expression in photosynthetic pathways of plants. Int. J. Mol. Sci..

[104] Nguyen D., Rieu I., Mariani C., van Dam N.M. (2016). How plants handle multiple stresses: Hormonal interactions underlying responses to abiotic stress and insect herbivory. Plant Mol. Biol..

[105] Verma V., Ravindran P., Kumar P.P. (2016). Plant hormone-mediated regulation of stress responses. BMC Plant Biol..

[106] Tao J.J., Chen H.W., Ma B., Zhang W.K., Chen S.Y., Zhang J.S. (2015). The role of ethylene in plants under salinity stress. Front. Plant Sci..

[107] Cheng W.H., Chiang M.H., Hwang S.G., Lin P.C. (2009). Antagonism between abscisic acid and ethylene in *Arabidopsis* acts in parallel with the reciprocal regulation of their metabolism and signaling pathways. Plant Mol. Biol..

[108] Ghassemian M., Nambara E., Cutler S., Kawaide H., Kamiya Y., McCourt P. (2000). Regulation of abscisic acid signaling by the ethylene response pathway in *Arabidopsis*. Plant Cell.

[109] Song S., Huang H., Gao H., Wang J., Wu D., Liu X., Yang S., Zhai Q., Li C., Qi T. (2014). Interaction between MYC2 and ETHYLENE INSENSITIVE3 modulates antagonism between jasmonate and ethylene signaling in *Arabidopsis*. Plant Cell.

[110] Breakspear A., Liu C., Roy S., Stacey N., Rogers C., Trick M., Morieri G., Mysore K.S., Wen J., Oldroyd G.E. (2014). The root hair “infectome” of *Medicago truncatula* uncovers changes in cell cycle genes and reveals a requirement for auxin signaling in rhizobial infection. Plant Cell.

[111] Staiger D., Brown J.W. (2013). Alternative splicing at the intersection of biological timing, development, and stress responses. Plant Cell.

[112] Dinesh-Kumar S.P., Baker B.J. (2000). Alternatively spliced N resistance gene transcripts: Their possible role in tobacco mosaic virus resistance. Proc. Natl. Acad. Sci. USA.

[113] Filichkin S.A., Priest H.D., Givan S.A., Shen R., Bryant D.W., Fox S.E., Wong W.K., Mockler T.C. (2010). Genome-wide mapping of alternative splicing in *Arabidopsis thaliana*. Genome Res..

[114] Laval V., Koroleva O.A., Murphy E., Lu C., Milner J.J., Hooks M.A., Tomos A.D. (2002). Distribution of actin gene isoforms in the *Arabidopsis* leaf measured in microsamples from intact individual cells. Planta.

[115] Cong L., Ran F.A., Cox D., Lin S., Barretto R., Habib N., Hsu P.D., Wu X., Jiang W., Marraffini L.A. (2013). Multiplex genome engineering using CRISPR/Cas systems. Science.

[116] Hsu P.D., Lander E.S., Zhang F. (2014). Development and applications of CRISPR-Cas9 for genome engineering. Cell.

[117] Moignard V., Macaulay I.C., Swiers G., Buettner F., Schutte J., Calero-Nieto F.J., Kinston S., Joshi A., Hannah R., Theis F.J. (2013). Characterization of transcriptional networks in blood stem and progenitor cells using high-throughput single-cell gene expression analysis. Nat. Cell Biol..

[118] Jaitin D.A., Weiner A., Yofe I., Lara-Astiaso D., Keren-Shaul H., David E., Salame T.M., Tanay A., van Oudenaarden A., Amit I. (2016). Dissecting immune circuits by linking CRISPR-pooled screens with single-cell RNA-seq. Cell.

[119] Datlinger P., Rendeiro A.F., Schmidl C., Krausgruber T., Traxler P., Klughammer J., Schuster L.C., Kuchler A., Alpar D., Bock C. (2017). Pooled CRISPR screening with single-cell transcriptome readout. Nat. Methods.

[120] Dixit A., Parnas O., Li B., Chen J., Fulco C.P., Jerby-Arnon L., Marjanovic N.D., Dionne D., Burks T., Raychowdhury R. (2016). Perturb-seq: Dissecting molecular circuits with scalable single-cell RNA profiling of pooled genetic screens. Cell.

[121] Wang T., Birsoy K., Hughes N.W., Krupczak K.M., Post Y., Wei J.J., Lander E.S., Sabatini D.M. (2015). Identification and characterization of essential genes in the human genome. Science.

[122] Altpeter F., Springer N.M., Bartley L.E., Blechl A.E., Brutnell T.P., Citovsky V., Conrad L.J., Gelvin S.B., Jackson D.P., Kausch A.P. (2016). Advancing crop transformation in the era of genome editing. Plant Cell.

[123] Cox D.B.T., Gootenberg J.S., Abudayyeh O.O., Franklin B., Kellner M.J., Joung J., Zhang F. (2017). RNA editing with CRISPR-Cas13. Science.

